# Disparity in the natural cycles of Borrelia burgdorferi and the agent of human granulocytic ehrlichiosis.

**DOI:** 10.3201/eid0502.990203

**Published:** 1999

**Authors:** M L Levin, F des Vignes, D Fish

**Affiliations:** Yale School of Medicine, New Haven, Connecticut 06520-8034, USA. Michael.Levin@Yale.edu

## Abstract

We studied the prevalence of Borrelia burgdorferi and the agent of human granulocytic ehrlichiosis (HGE) among questing nymphal and adult Ixodes scapularis ticks of the same generation and the infectivity of wild white-footed mice for ticks feeding on them. The prevalence of B. burgdorferi infection in host-seeking ticks increased less than twofold from nymphal (31% to 33%) to adult (52% to 56%) stage, and 52% of white-footed mice were infected. Prevalence of the agent of HGE increased 4.5- to 10.6-fold from nymphal (1.5% to 1.8%) to adult stage (7.6% to 19.0%), while only 18% of mice were infectious to ticks. B. burgdorferi infection was more common in mouse-fed ticks than in ticks collected from vegetation, whereas the agent of HGE was half as common in mouse-fed ticks as in ticks collected from vegetation. The different prevalence in nature of these pathogens in ticks suggests that their maintenance cycles are also different.


### Disparity in the Natural Cycles of *Borrelia burgdorferi* and the Agent of Human Granulocytic Ehrlichiosis

**Michael L. Levin, Franka des Vignes, and Durland Fish**
Yale School of Medicine, New Haven, Connecticut, USA

We studied the prevalence of *Borrelia burgdorferi* and the agent of human granulocytic ehrlichiosis (HGE) among questing nymphal and adult *Ixodes scapularis* ticks of the same generation and the infectivity of wild white-footed mice for ticks feeding on them. The prevalence of *B. burgdorferi* infection in host-seeking ticks increased less than twofold from nymphal (31% to 33%) to adult (52% to 56%) stage, and 52% of white-footed mice were infected. Prevalence of the agent of HGE increased 4.5- to 10.6-fold from nymphal (1.5% to 1.8%) to adult stage (7.6% to 19.0%), while only 18% of mice were infectious to ticks. *B. burgdorferi* infection was more common in mouse-fed ticks than in ticks collected from vegetation, whereas the agent of HGE was half as common in mouse-fed ticks as in ticks collected from vegetation. The different prevalence in nature of these pathogens in ticks suggests that their maintenance cycles are also different.

The agent of human granulocytic ehrlichiosis (HGE) is nearly identical to *Ehrlichia phagocytophila*, which causes tick-borne fever in sheep and goats in Europe (1,2) and is transmitted by the tick *Ixodes ricinus* (3)—the major vector of Lyme disease (4). In the eastern and midwestern United States, the agent of HGE is transmitted by *I. scapularis* (5-7), also a vector of *Borrelia burgdorferi*—the agent of Lyme disease. Both agents are perpetuated in natural cycles between the tick vector and vertebrate hosts (8-10). Neither agent is maintained transovarially (11,12); thus, horizontal transmission involving a susceptible vertebrate host is necessary. The white-footed mouse (*Peromyscus leucopus*) plays an important role as a reservoir for *B. burgdorferi* (13). Animal species that serve as the main source(s) of the agent of HGE for ticks have not been determined.

Rodents serving as natural hosts for the tick species that transmits both agents can be coincidentally exposed to the two agents. Indeed, white-footed mice from Connecticut have been shown to carry antibodies to both agents simultaneously (14). Granulocytic ehrlichia have been found in wild rodents (10,15). Furthermore, the white-footed mouse and several strains of laboratory mice (*Mus musculus)* are susceptible to the agent of HGE in laboratory experiments (5,10,12). Antibodies to the agent of HGE have been detected in various rodent species from California, Colorado, Connecticut, Florida, New Jersey, New York, Maryland, Minnesota, and Wisconsin (14,16-18). These findings allowed the authors to suggest that small rodents, particularly the white-footed mouse, play the same role in perpetuating the agent of HGE in North America that they do in perpetuating *B. burgdorferi* (10,14,16,18,19). We present results of a 2-year field study that provide evidence to the contrary.

### The Study

We studied the prevalence of *B. burgdorferi* and the agent of HGE among questing nymphal and adult *I. scapularis* of the same generation, in natural foci with concurrent circulation of both pathogens in Connecticut. In 1996, we collected ticks at two study sites—Bridgeport and Woodbridge—approximately 30 km apart. In 1997, we collected ticks at the Bridgeport site

Address for correspondence: Michael L. Levin, Department of Epidemiology and Public Health, Yale School of Medicine, 60 College Street, #600, P.O. Box 208034, New Haven, CT, 06520-8034, USA; fax: 203-785-4782; e-mail: Michael.Levin@Yale.edu.

only; ticks feeding on white-footed mice and from vegetation were collected and flagged in June (nymphs) and October (adults). Mice were trapped in Sherman live-traps twice per month from June through August and were held for several days in the laboratory in individual wire-mesh cages over water to allow all naturally attached ticks to feed to repletion. While in the laboratory, mice were provided with food and drinking water *ad libitum*. Engorged ticks were collected daily. A serum sample was collected from each mouse before release at the capture site.

Serum samples from 121 mice were tested for specific antibodies to the agent of HGE by indirect immunofluorescence assay (IFA) (Aquila Biopharmaceuticals, Worcester, MA). Antigen derived from human promyelocyte cell culture (HL-60) infected with the agent of HGE obtained from Westchester County, New York. Sera were screened at a dilution of 1:40 in 1X phosphate-buffered saline (pH 7.4). Several studies examined the specificity of IFA for the agent of HGE (including an assay involving the antigen produced by Aquila Biopharmaceuticals) and found no considerable serologic cross-reactivity between the agent of HGE and rickettsial organisms outside the *E. phagocytophila* group at dilutions 1:16 and higher (8,20,21). Thus, screening samples at 1:40 dilution ensures that sera testing HGE-positive were from mice actually exposed to that agent.

Host-seeking nymphal and adult *I. scapularis* collected from vegetation were tested individually by polymerase chain reaction (PCR) for infection with *B. burgdorferi* and the agent of HGE. Engorged ticks were allowed to molt to the next stage and were identified by species. *I. scapularis* nymphs derived from mouse-fed larvae were tested in pools—one pool (up to 10 ticks) per mouse—to assess the infectivity of individual mice for feeding larvae. Adult *I. scapularis* ticks derived from mouse-fed nymphs were tested individually, and the prevalence of each infection among these ticks was compared with the prevalence of each infection among questing adult ticks collected from vegetation at the same site.

For PCR testing, individual adult or nymphal ticks, or pools of nymphs, were placed in sterile 1.5 cc plastic vials, deep-frozen in liquid nitrogen, ground with a sterile plastic pestle, and resuspended in 50 ml of Tris-EDTA buffer. DNA was extracted from samples by using the IsoQuick Nucleic Acid Extraction Kit (ORCA Research Inc., Bothell, WA) to maximize sensitivity (22). Briefly, guanidine thiocyanate, a proprietary extraction matrix, and sodium dodecyl sulfate solution were added to a suspension, and the mixture was incubated at 65°C for 10 min. After separation of phases by centrifugation, DNA was precipitated with sodium acetate and isopropanol and washed with 70% ethanol. The final DNA pellet was resuspended in 50 ml of RNase-free water, and a 2.5-ml aliquot was used for each PCR. Primers EHR521 (5'-TGT AGG CGG TTC GGT AAG TTA AAG-3') and EHR747 (5'-GCA CTC ATC GTT TAC AGC GTG-3') were used to amplify a 247-bp fragment of 16S rDNA from the agent of HGE (6). Primers FLA297 (5'-CGG CAC ATA TTC AGA TGC AGA CAG-3') and FLA652 (5'-CCT GTT GAA CAC CCT CTT GAA CC-3') developed in the laboratory of Dr. Erol Fikrig (Yale School of Medicine) were used to amplify a 378-bp fragment of the flagellin gene of *B. burgdorferi*. The amplification products were visualized in 2% agarose gels.

### Prevalence of Infections

The prevalence of *B. burgdorferi* infection in both questing nymphal and adult *I. scapularis* was similar between the two study sites in 1996 and remained stable for 2 consecutive years at the Bridgeport site (Table 1). Within a generation, the proportion of *Borrelia*-infected ticks increased less than twofold from nymphal (32% to 33%) to adult stage (52% to 56%) at both sites and in both years.

Prevalence of ehrlichial infection in *I. scapularis* nymphs was also similar at the two study sites and between years at the Bridgeport site (Table 1), while in questing adult ticks, ehrlichial prevalence varied. In 1996, the

Table 1. Prevalence of infection with *Borrelia burgdorferi* and the agent of human granulocytic ehrlichiosis among questing nymphal and adult *Ixodes scapularis* of the same generation, Connecticut

| Study site (year) | Stage | Ticks tested | % *Borrelia*-infected (± SE) | % *Ehrlichia*-infected (± SE) |
|---|---|---|---|---|
| Woodbridge | Nymphs | 442 | 32.9 ± 2.3 | 1.6 ± 0.6 |
| (1996) | Adults | 251 | 52.6 ± 3.2 | 8.3 ± 1.9 |
| Bridgeport | Nymphs | 164 | 31.7 ± 2.2 | 1.2 ± 0.5 |
| (1996) | Adults | 48 | 56.3 ± 3.2 | 12.5 ± 2.1 |
| Bridgeport | Nymphs | 110 | 32.7 ± 4.5 | 1.8 ± 1.3 |
| (1997) | Adults | 100 | 55.0 ± 3.8 | 19.0 ± 3.0 |

prevalence of the agent of HGE in adult ticks at the Bridgeport site (12.5%) was higher than at the Woodbridge site (8.3%), although not significantly ($p = 0.11$). The proportion of adult ticks infected with the agent of HGE in Bridgeport was significantly higher in 1997 than in the previous year ($p = 1.44 \times 10^{-7}$). Within the same generation, the proportion of *Ehrlichia*-infected ticks increased fivefold in Woodbridge in 1996 and 10.4- to 10.6-fold in Bridgeport in 1996 and 1997, respectively.

An average of 0.7 engorged *I. scapularis* nymphs (0 to 7) and 16.7 (0 to 113) engorged *I. scapularis* larvae were collected per mouse. The highest mean density (± standard error) of nymphal infestation in *P. leucopus* (1.3±0.2) was recorded in late June, with mean larval density peaking in late August (34.6±1.7). Overall, 113 of 121 mice were infested with either nymphal or larval *I. scapularis.* Of 40 mice with nymphs, 32 (80.0%) produced adult ticks infected with *B. burgdorferi*, and 7 (17.5%) yielded ticks infected with the ehrlichial agent. Of 108 mice infested with *I. scapularis* larvae, 56 (51.4%) produced *B. burgdorferi*-infected nymphal ticks, and 20 (18.4%) produced nymphs infected with the agent of HGE. Of the 108 mice, 13 (11.9%) infected feeding ticks with both pathogens. Prevalence of infectivity in mice did not differ by month.

Of 121 mice trapped from June to August 1997 in Bridgeport and tested by IFA, 46 (38.0%) had antibodies against the agent of HGE. The relatively high sera dilution (1:40) used for screening allows the possibility that some samples with low antibody titers were missed and that an even higher proportion of the mouse population had actually been exposed to the agent of HGE.

The proportion of HGE-seropositive mice decreased from 47% (n = 49) in late June and July to 32% (n = 72) in August, perhaps because of a loss of antibody by mice over time, the recruitment of naive young mice into the population, or both. Of the 20 mice that produced nymphs infected with the agent of HGE, 17 were also seropositive. Evidently, these mice remained infectious for ticks, despite the presence of specific antibodies. The other three infectious mice were HGE-seronegative, which most likely indicated recent infection. Thus, only 18.4% of *P. leucopus* were capable of infecting ticks with the agent of HGE, although at least twice as many (as determined by IFA) had been exposed to this agent. Apparently, mice exposed to ticks infected with the agent of HGE may develop an immune response to the pathogen but not become infectious for xenodiagnostic ticks. These findings suggest that a high prevalence of the specific antibody against the agent of HGE in rodent populations does not necessary reflect the scope of rodents' involvement in transmitting the ehrlichial agent.

Prevalence of *B. burgdorferi* infection among adult ticks derived from mouse-fed nymphs was higher than among questing ticks collected from vegetation ($p = 0.0051$) (Table 2). Conversely, prevalence of infection with the agent of HGE in mouse-fed ticks was not quite half that of adult ticks collected from vegetation ($p = 0.0022$). Concurrent infection in mouse-fed ticks was also half that in adult ticks collected from vegetation (Table 2). The same trend was observed when prevalence of ehrlichial infection was compared among *Borrelia*-infected adult ticks. In the *Borrelia*-infected cohort collected from vegetation, 14 (25.5%) of 55 ticks were simultaneously infected with the agent of HGE, but in the cohort collected from mice, only 5 (10.2%) of 49 were simultaneously infected with the agent of HGE.

Mice that collect and feed many nymphs have a high probability of finding an infected tick and becoming infected. Mice that feed large numbers of ticks after infection would increase the rate of pathogen transmission. We tested the hypothesis that mice exposed to or currently infected with the agent of HGE are continuously infested with large numbers of ticks and thus are capable of increasing the prevalence of infection in a natural tick population. When we compared tick densities between mice infectious for ticks, mice seropositive for the agent of HGE, and mice

Table 2. Prevalence of infection with *Borrelia burgdorferi* and the agent of human granulocytic ehrlichiosis among adult *Ixodes scapularis* ticks collected as engorged nymphs from wild-caught *Peromyscus leucopus* and from vegetation at the same site (Bridgeport, Connecticut)

| Origin of ticks | Ticks tested | % *Borrelia* infected (± SE) | % *Ehrlichia*-infected (± SE) | % Concurrently infected (± SE) |
|---|---|---|---|---|
| *P. leucopus* | 76 | 64.5 ± 3.6 | 9.2 ± 2.2 | 6.6 ± 2.5 |
| Vegetation | 100 | 55.0 ± 3.8 | 19.0 ± 3.0 | 14.0 ± 3.5 |

without specific antibodies or infected ticks, nymphal and larval infestation densities did not differ significantly (Table 3).

Table 3. Exposure to the agent of human granulocytic ehrlichiosis in white-footed mice, as detected by xenodiagnosis and indirect immunoflorescence assay (IFA), and their infestation with *Ixodes scapularis* nymphs and larvae (Bridgeport, Connecticut)

| Status of mice | Mice tested | Mean no. of nymphs (± SE) | Mean no. of larvae (± SE) |
|---|---|---|---|
| Infectious[a] | 20 | 0.7±0.1 | 19.5±1.6 |
| IFA-positive | 46 | 0.9±0.1 | 14.9±0.9 |
| IFA-negative, noninfectious | 72 | 0.7±0.1 | 17.8±1.9 |

[a]Mice infectious for ticks at the time of study.

### Conclusions

Because nymphal *I. scapularis* naturally feed on vertebrate animals (small rodents, medium-sized and large mammals, birds, and reptiles) (23) that may vary in their ability to acquire and transmit a pathogen, the prevalence of infection in different portions of an adult tick population would differ depending on which host species the tick had fed on. Thus, the prevalence of infection for the tick population as a whole is an average resulting from contributions of individual host species. We compared average prevalence to the prevalence of infection in ticks derived from a particular host species to assess the relative contribution of that host species to amplification of a pathogen. For example, the prevalence of *B. burgdorferi* infection in mouse-fed ticks is considerably higher than the average prevalence of the infection in a general tick population, which suggests that the white-footed mouse is an important amplifying reservoir of *B. burgdorferi*. The prevalence of the agent of HGE in mouse-fed ticks is lower than the average, suggesting that the white-footed mouse is not as effective an amplifying reservoir for this agent as are other host species. An important contribution from non-*Peromyscus* host species that infects a large proportion of feeding nymphs with the agent of HGE appears necessary to account for the average prevalence of infection in host-seeking adult ticks.

The prevalence of *B. burgdorferi* infection in ticks increased less than twofold from nymphal to adult stage, while more than 50% of the white-footed mouse population was infected with *B. burgdorferi* and transmitted the pathogen to feeding ticks. At the same time, prevalence of infection with the agent of HGE in ticks regularly showed a 4.5- to 10.6-fold increase from nymphal stage to adult stage, although only 18% of mice were infectious for feeding ticks. This steep increase in prevalence of ehrlichial infection in ticks also suggests the involvement of other susceptible host species that maintain the natural transmission cycle of the agent of HGE at the observed level.

Dissimilarities between two pathogens—in the increase of infection in ticks from nymphal to adult stage and in prevalence of infection in the host-seeking population versus a subpopulation of mouse-fed ticks—suggest that natural cycles of the agents of Lyme disease and HGE differ. They involve the same vector-species, but the principal amplifying hosts for the two pathogens are not the same.

Although the white-footed mouse is susceptible to infection with both agents, this species alone cannot account for the observed prevalence of the agent of HGE in adult ticks. Our data suggest that most nymphal *I. scapularis* acquire the agent of HGE from non-*Peromyscus* hosts.

### Acknowledgments

We thank Yukiko Otsuka for assistance in both the field and laboratory and Richard Coughlin for providing IFA slides for our assays.

This research was sponsored by grants from G. Harold and Leila Y. Mathers Charitable Foundation, the National Institutes of Health, National Institute of Allergy and Infectious Diseases (AI28956), and U.S. Department of Agriculture cooperative agreement 58-1265-5023.

Dr. Levin is a postdoctoral associate, Department of Epidemiology and Public Health, Yale School of Medicine. His areas of expertise are ecology and epidemiology of tick-borne zoonoses. His current research focuses on the dynamics of circulation of the agents of Lyme disease and human granulocytic ehrlichiosis in nature, reservoir competence of vertebrate animals, and multiple pathogen interactions.

### References

1. Gordon WS, Brownlee A, Wilson DR, MacLeod J. "Tick-borne fever": a hitherto undescribed disease of sheep. Journal of Comparative Pathology and Therapy 1932;65:301-7.
2. Sumner JW, Nicholson WL, Massung RF. PCR amplification and comparison of nucleotide sequences from the groESL heat shock operon of *Ehrlichia* species. J Clin Microbiol 1997;35:2087-92.

3. MacLeod JR, Gordon WS. Studies of tick-borne fever of sheep. 1. Transmission by the tick *Ixodes ricinus*, with a description of the disease produced. Parasitology 1933;25:273-85.
4. Ackermann R. The spirochetal etiology of erythema chronicum migrans and of meningo-polyneuritis Garin-Bujadoux-Bannwarth. [German]. Zeitschrift fur Hautkrankeiten 1983;58:1616-21.
5. Des Vignes F, Fish D. Transmission of the agent of human granulocytic ehrlichiosis by host-seeking *Ixodes scapularis* (Acari:Ixodidae) in southern New York state. J Med Entomol 1997;34:379-82.
6. Pancholi P, Kolbert CP, Mitchell PD, Reed KD Jr, Dumler JS, Bakken JS, et al. *Ixodes dammini* as a potential vector of human granulocytic ehrlichiosis. J Infect Dis 1995;172:1007-12.
7. Schwartz I, Fish D, Daniels TJ. Prevalence of the rickettsial agent of human granulocytic ehrlichiosis in ticks from a hyperendemic focus of Lyme disease. N Engl J Med 1997;337:49-50.
8. Magnarelli LA, Stafford KC, Mather TN, Yeh MT, Horn KD, Dumler JS. Hemocytic rickettsia-like organisms in ticks: serologic reactivity with antisera to *Ehrlichiae* and detection of DNA of agent of human granulocytic ehrlichiosis by PCR. J Clin Microbiol 1995;33:2710-4.
9. Reed KD, Mitchell PD, Persing DH, Kolbert CP, Cameron V. Transmission of human granulocytic ehrlichiosis [letter; comment]. JAMA 1995;273:23.
10. Telford SR, Dawson JE, Katavolos P, Warner CK, Kolbert CP, Persing DH. Perpetuation of the agent of human granulocytic ehrlichiosis in a deer tick-rodent cycle. Proc Natl Acad Sci U S A 1996;93:6209-14.
11. Patrican LA. Absence of Lyme disease spirochetes in larval progeny of naturally infected *Ixodes scapularis* (Acari:Ixodidae) fed on dogs. J Med Entomol 1997;34:52-5.
12. Hodzic E, Fish D, Maretzki CM, de Silva AM, Feng S, Barthold SW. Acquisition and transmission of the agent of human granulocytic ehrlichiosis by *Ixodes scapularis* ticks. Infect Immun. J. Clin Microbiol 1998;36:3574-8.
13. Barbour AG, Fish D. The biological and social phenomenon of Lyme disease [review]. Science 1993;260:1610-6.
14. Magnarelli LA, Anderson JF, Stafford KC, Dumler JS. Antibodies to multiple tick-borne pathogens of babesiosis, ehrlichiosis, and Lyme disease in white-footed mice. J Wildl Dis 1997;33:466-73.
15. Tyzzer EE. *Cytoecetes microti* n. gen. n. sp.: a parasite developing in granulocytes and infection in small rodents. Parasitology 1938;30:242-57.
16. Walls JJ, Greig B, Neitzel DF, Dumler JS. Natural infection of small mammal species in Minnesota with the agent of human granulocytic ehrlichiosis. J Clin Microbiol 1997;35:853-5.
17. Bunnell JE, Dumler JS, Childs JE, Glass GE. Retrospective serosurvey for human granulocytic ehrlichiosis agent in urban white-footed mice from Maryland. J Wildl Dis 1998;34:179-81.
18. Nicholson WL, Muir S, Sumner JW, Childs JE. Serologic evidence of infection with *Ehrlichia* spp. in wild rodents (Muridae:Sigmodontinae) in the United States. J Clin Microbiol 1998;36:695-700.
19. Daniels TJ, Falco RC, Schwartz I, Varde S, Robbins RG. Deer ticks (*Ixodes scapularis*) and the agents of Lyme disease and human granulocytic ehrlichiosis in a New York City park. Emerg Infect Dis 1997;3:353-5.
20. Dumler JS, Asanovich KM, Bakken JS, Richter P, Kimsey R, Madigan JE. Serologic cross-reactions among *Ehrlichia equi*, *Ehrlichia phagocytophila*, and human granulocytic Ehrlichia. J Clin Microbiol 1995;33:1098-103.
21. Nicholson WL, Comer JA, Sumner JW, Gingrichbaker C, Coughlin RT, Magnarelli LA, et al. An indirect immunofluorescence assay using a cell culture-derived antigen for detection of antibodies to the agent of human granulocytic ehrlichiosis. J Clin Microbiol 1997;35:1510-6.
22. Schwartz I, Varde S, Nadelman RB, Wormser GP, Fish D. Inhibition of efficient polymerase chain reaction amplification of *Borrelia burgdorferi* DNA in blood-fed ticks. Am J Trop Med Hyg 1997;56:339-42.
23. Anderson JF. Epizootiology of Lyme borreliosis [review]. Scand J Infect Dis Suppl 1991;77:23-34.
